# The Diversity of Muscles and Their Regenerative Potential across Animals

**DOI:** 10.3390/cells9091925

**Published:** 2020-08-19

**Authors:** Letizia Zullo, Matteo Bozzo, Alon Daya, Alessio Di Clemente, Francesco Paolo Mancini, Aram Megighian, Nir Nesher, Eric Röttinger, Tal Shomrat, Stefano Tiozzo, Alberto Zullo, Simona Candiani

**Affiliations:** 1 Istituto Italiano di Tecnologia, Center for Micro-BioRobotics & Center for Synaptic Neuroscience and Technology (NSYN), 16132 Genova, Italy; Alessio.DiClemente@iit.it; 2 IRCCS Ospedale Policlinico San Martino, 16132 Genova, Italy; 3 Laboratory of Developmental Neurobiology, Department of Earth, Environment and Life Sciences, University of Genova, Viale Benedetto XV 5, 16132 Genova, Italy; matteo.bozzo@edu.unige.it (M.B.); candiani@unige.it (S.C.); 4 Faculty of Marine Sciences, Ruppin Academic Center, Michmoret 40297, Israel; alond@ruppin.ac.il (A.D.); nirn@ruppin.ac.il (N.N.); talsh@ruppin.ac.il (T.S.); 5 Department of Experimental Medicine, University of Genova, Viale Benedetto XV, 3, 16132 Genova, Italy; 6 Department of Science and Technology, University of Sannio, 82100 Benevento, Italy; mancini@unisannio.it; 7 Department of Biomedical Sciences, University of Padova, 35131 Padova, Italy; aram.megighian@unipd.it; 8 Padova Neuroscience Center, University of Padova, 35131 Padova, Italy; 9 Institute for Research on Cancer and Aging (IRCAN), Université Côte d’Azur, CNRS, INSERM, 06107 Nice, France; eric.rottinger@univ-cotedazur.fr; 10 Laboratoire de Biologie du Développement de Villefranche-sur-Mer (LBDV), Sorbonne Université, CNRS, 06230 Paris, France; tiozzo@obs-vlfr.fr

**Keywords:** myogenesis, evolution, metazoans, differentiation, transdifferentiation, muscle precursors, regenerative medicine

## Abstract

Cells with contractile functions are present in almost all metazoans, and so are the related processes of muscle homeostasis and regeneration. Regeneration itself is a complex process unevenly spread across metazoans that ranges from full-body regeneration to partial reconstruction of damaged organs or body tissues, including muscles. The cellular and molecular mechanisms involved in regenerative processes can be homologous, co-opted, and/or evolved independently. By comparing the mechanisms of muscle homeostasis and regeneration throughout the diversity of animal body-plans and life cycles, it is possible to identify conserved and divergent cellular and molecular mechanisms underlying muscle plasticity. In this review we aim at providing an overview of muscle regeneration studies in metazoans, highlighting the major regenerative strategies and molecular pathways involved. By gathering these findings, we wish to advocate a comparative and evolutionary approach to prompt a wider use of “non-canonical” animal models for molecular and even pharmacological studies in the field of muscle regeneration.

## 1. Introduction

One particular challenge in regenerative biology concerns the development of reconstructive strategies after muscle-related injuries, but also the treatments of degenerative myopathies for which no reliable clinical strategy exists such as muscle dystrophy, sarcopenia, cachexia, to mention just a few [1,2]. In mammals regenerative capacities are restricted to only a small number of organs [3], yet, in other metazoans, the ability to respond to environmental injuries ranges from “simple” wound healing to complete anatomical and functional restoration of the lost or damaged part of the body, including muscles [4]. The musculature is a tissue specialized in contraction and cells with contractile functions are present in almost all metazoans but, despite their structural similarity, the origin of muscles is considered to be the outcome of a process of convergent evolution [5]. Indeed, typical muscle protein core sets are present even in unicellular organisms and in early diverged organisms like sponges, which lacks a proper tissue organization and therefore “true” muscles, and in cnidarians, where muscle-like cells are present but lack almost all molecular hallmarks of bilaterian striated muscles thus suggesting evolution from cells with ancient contractile machinery [5]. The processes of myogenesis and muscle homeostasis have also various degrees of conservation among different clades, and so is the extent of muscle regenerative capabilities [5,6,7,8,9,10].

Animals adopt different basic strategies of regeneration that include the activation of adult stem cells, the dedifferentiation of preexisting cells, and/or the proliferation of differentiated cells. This diversity of mechanisms is still widely understudied and underexploited for biomedical applications.

**Figure 1:**
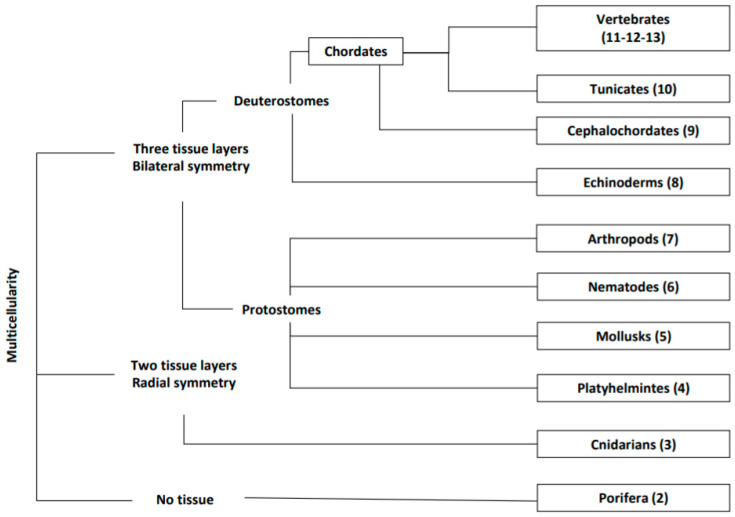
Tree of the animals’ clades treated in this review (in brackets the corresponding section numbers).

In this review, we provide an outline of main animals’ clades (see Figure 1), muscle types, their development, homeostasis, and regeneration abilities highlighting what is known of their molecular mechanisms. We emphasize some potential contributions of comparative studies into the biomedical fields, therefore advocating deeper employment of ‘non-canonical’ animals as models for muscle regeneration studies.

Understanding the molecular pathways and mechanistic underlying regenerative events may offer insights into potential methods to unlock regeneration in animals where the regenerating capabilities are more restricted, e.g., mammals. Indeed, regeneration is greatly attenuated in mammals although portions of major organs, such as the liver, retain event-triggered regenerative potentials for the entire animal’s life [2]. Interestingly, recent pieces of evidence suggest that regeneration can be induced even in non-regenerating species by altering specific signaling pathways [11,12,13]. This might be also the case for mammals and thus the principles underlying the induction of regeneration in non-regenerating species may be transferred to humans to trigger regeneration [3,14].

Elucidating muscle regeneration in metazoans also provides opportunities to ‘model’ a complex biological process relevant to human health and offers a window into fundamental principles underpinning this important response.

## 2. Porifera: Low Body Complexity with High Regenerative Capabilities

The phylum Porifera includes mainly sponges, aquatic multicellular organisms with relatively simple anatomy, lacking an organization of tissues and organs.

They have a very simple functioning relying on water circulating throughout a system of canals and chambers, called a water-current system. Circulatory, digestive, nervous, and muscular systems are completely absent. Their body is composed of a few types of cells [15,16]. For instance, in demosponges we found pinacocytes (the skin cells), mesenchymal cells, choanocytes (lining in the interior body walls), archaeocytes (totipotent cells), sclerocytes, myocytes, and porocytes (surrounding canal openings). Two types of contractile cells can be identified: the pinacocytes, and the myocytes. Pinacocytes form a functional contractile epithelium. They are composed of actin networks and actin-dense plaques allowing a coordinated contraction in adjacent pinacocytes but their mechanism of contractility remains to be further elucidated [17].

Most sponge species have an extraordinary capacity to regenerate lost body parts. Four cell types have been identified as stem-cell-like in sponge: choanocytes and archaeocytes, also referred to as adult stem cells (ASCs), pinacocytes, and particular ameboid vacuolar cells [18,19].

### Muscle-Like Cells

Myocytes are spindle-shaped, smooth muscle-like cells containing microtubules lying parallel to peripheral microfilaments. They contract similarly to muscle cells thanks to a non-muscle myosin type II with high homology to that found in bilaterians and vertebrates. They allow the sponge to change shape and expel sediments even without the presence of a nervous system as their contraction is entirely controlled at a cellular level through variation of calcium (Ca^2+^) concentration [20,21]. In particular, channels located at the plasma membrane allow the control of intracellular Ca^2+^ concentration that, in turn, regulates cell contractility. This mechanism is believed to be rely on the activation of type II myosin by Ca^2+^-dependent protein kinases [20,22]. Myocytes allow only movement internal to the sponge but these animals remain essentially sessile. Predation and physical injuries are events very common during the entire adult life of sessile organisms that had to develop efficient strategies of repair and replacement of lost structures to survive [23,24].

Very limited information is currently available on the molecular pathways involved in body regeneration. The activity of ADP-ribosyl cyclase (ADPRC) is related to physiological activities in sponges, such as stem cell duplication and regeneration events [25]. Sponges regenerate using diverse and complex morphogenetic mechanisms involving different cell sources depending on the species. Regeneration can occur through epimorphosis and morphallaxis. The first process involves the formation of a mass of undifferentiated cells (blastema) at the wound site. Pluripotent cells such as archaeocytes and choanocytes from sites adjacent to the injury, undergo a process of epithelial-to-mesenchymal transition (EMT) and migrate to the injured area. Here they actively proliferate and form a typical blastema with dedifferentiated cells. Thereafter a process of mesenchymal-to-epithelial transition (MET) re-establish the differentiated cell identity. Different members of the transforming growth factor (TGF) superfamily are also involved in these processes [26]. In morphallaxis, of particular importance is cell transdifferentiation, the conversion of a differentiated cell to another type of differentiated cell [18]. During this process, spreading and fusion of the epithelia surrounding the wound is accompanied by the transdifferentiation of the choanocytes and exopinacocytes without the formation of a blastema. This supports the hypothesis that these cells combine properties of somatic and stem cells.

Taken together, Porifera represents, for their exceptional regenerative capacities and low body complexity, a promising model for investigating mechanisms of cell recognition, adhesion, migration, and cell type transition during regeneration.

## 3. Cnidarians: The Starlet Sea Anemone, *Nematostella vectensis* (Anthozoa)

**Figure 2:**
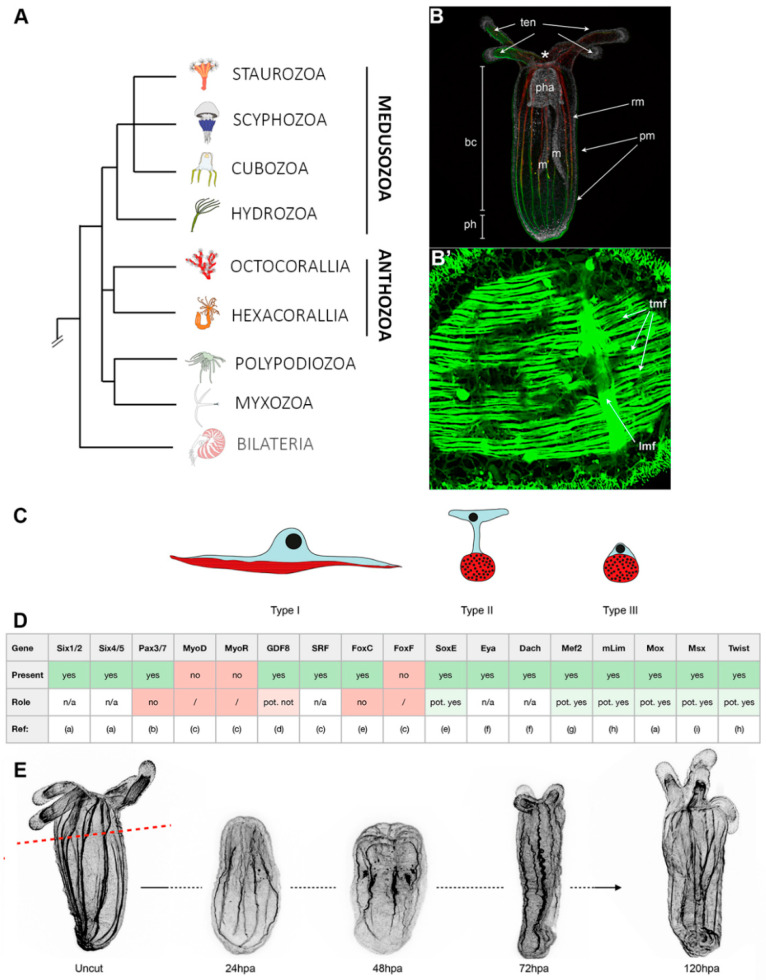
The epitheliomuscular system and regenerative capacity of the anthozoan cnidarian *Nematostella vectensis*. (**A**) Schematic representation of the relationship between the main cnidarian lineages and the phylogenetic position of *Nematostella vectensis* (Anthozoa, Hexacorallia). (**B**) The upper panel shows the muscle network of nematostella in a fixed MyHC1::mCherry transgene [30] labeling the retractor muscles, co-stained with phalloidin thus showing the entire muscle network in green. (ten) tentacles, (*) mouth, (pha) pharynx, (bc) body column, (ph) physa, (m) mesenteries, (rm) retractor muscles, (pm) parietal muscles. (**B’**) Magnification of a body column region to highlight the orientation of the muscle fibers. (tmf) transverse muscle fibers, (lmf) longitudinal muscle fibers. (**C**) Three epitheliomuscular cell types have been identified in nematostella; they vary in their apical and basal cell junctions as well as their localizations within the body [31]. (**D**) Overview of the known bilateral myogenic factors identified in nematostella. (Present) indicates that the gene has been identified in the genome, (Role) indicates a myogenic role (or not) of this gene in nematostella; (pot. yes), indicates evidence of a myogenic role based on functional experiments or gene expression. (pot. not), indicates evidence of a non-myogenic role based on functional experiments or gene expression. (n/a) data not available. References cited: (a) [32], (b) [33], (c) [34], (d) [35], (e) [36], (f) [37], (g) [38], (h) [39], (i) [40]. (**E**) Oral regeneration of lost body parts after sub-pharyngeal amputation (red dashed line) is completed after 120 h post amputation and reforms a fully functional organism. Animals were fixed at various time points during regeneration and stained with phalloidin to show f-actin filaments (black). Elements of the figure are extracted from [28,41].

Cnidarians (*Hydra*, jellyfish, corals, sea anemones) are aquatic animals that are the sister group to the bilaterian clade [27] and hold a key phylogenetic position for understanding the evolution of common biological processes and mechanisms [28]. The two main groups of the phylum Cnidaria are Medusozoa (jellyfish, hydroids, *Hydra*) and Anthozoa (corals, sea anemones) (Figure 2A). Cnidarians are structurally simple animals with remarkable regeneration capacity. They can regenerate amputated head, foot, and intact animals can even regenerate grouping single dissociated cells [29].

The present section focuses on the sea anemone *Nematostella vectensis* that belongs to the Anthozoa, mostly sessile cnidarians that are represented by individual or colony-forming polyps.

The sea anemone *Nematostella vectensis* (Anthozoa, Figure 2B), was initially employed for studying the evolution of embryonic developmental mechanisms [42] and is now emerging as a novel complementary whole-body regeneration model [41]. Nematostella possesses a range of fundamental advantages, such as the access to biological material, a relatively short life-cycle, an annotated genome that revealed astonishing similarities with the one from vertebrates [34], a wealth of -omics data [43,44] and well developed functional genomics and genome editing approaches [45,46,47].

Nematostella is a rather small sea anemone (juveniles ~0.5 mm, adults ~3 cm), translucent, and well suited for imaging purposes (Figure 2B). It is a diploblastic animal formed by a bifunctional internal endomesoderm and an outer ectoderm. On the oral extremity are the tentacles that surround the mouth and the so-called physa on the opposite. Food caught by the tentacles is ingested via a muscular and neuron-rich pharynx and digested within the body cavity. While most of the digestive enzymes are secreted by the mesenteries that also store nutrients [37], these internal structures play another role as they harbor the gonads that are crucial for sexual reproduction [42] and for inducing a regenerative response [48].

### 3.1. Muscle Types, Organization, and Myogenic Genes

Cnidarians display a large diversity of muscle types and organizations that are involved in multiple crucial physiological functions such as feeding, locomotion, or defense [28]. Although this group of marine invertebrates lacks a large part of the molecular hallmarks of striated muscles [5], jellyfish present some ultrastructural and functional features (such as striated myonemes, thick and thin myofilaments, desmosomes as well as a mechanism of excitation–contraction coupling based on intracellular calcium stores [49]) resembling the structure and function of striated muscles [50,51,52,53].

For a global overview of cnidarian muscle diversity, their development, and regeneration, please refer to [28]. Most anthozoan muscle cells, and nematostella is no exception, are epitheliomuscular; they contain smooth myofilaments [28] forming a transverse and longitudinal muscle fiber network clearly visible using a MyHC1::mCherry transgenic line [30] and phalloidin/actin staining (Figure 2B’). The epitheliomuscular cells, whose actin fibers form more or less condensed muscle fibers are responsible for various functions of the animal such as feeding or locomotion.

A recent study has characterized three epitheliomuscular cell types (Figure 2C); two types (I and II) with elongated cytoplasmic bridges present in the endodermal parietal and retractor muscles (Figure 2B) and one type III corresponding to basiepithelial muscle cells encountered in the ectoderm of the tentacles [31]. While the known bilaterian myogenic regulatory factors (MRFs [54]) are missing in nematostella (e.g., MyoD, MyoR), a large part of the conserved myogenic gene regulatory network (e.g., Pax3, Pax7, Six1 [55]) has been identified (Figure 2D, reviewed in [28]). However, their exact roles in the formation of the epitheliomuscular cells in nematostella are yet to be understood.

### 3.2. Muscle Regeneration and Role of Epitheliomuscular Cells in the Regenerative Process

Like cnidarians in general, nematostella possesses proliferation-dependent whole-body regenerative capacities and can regrow fully functional animals from isolated body pieces within less than a week (Figure 2E) [56,57,58,59]. In addition, nematostella is very well suited to compare embryonic development and whole-body regeneration within the same organism [43,57,60], one of the long-lasting questions in regenerative biology [61].

By assessing the MyHC1::mCherry transgenic gene expression after bisection, Renfer and colleagues have observed that the retractor muscles retract from the wound site immediately after amputation. In later steps of the regenerative process, mCherry positive cells accumulate in the regenerating body part suggesting active cellular re-organization and differentiation to reform the retractor muscles [30]. However, the cellular and molecular mechanisms underlying retractor muscle regeneration remain unknown.

On the other hand, there are shreds of evidence suggesting an active role of muscle fibers in the regenerative process. Bossert and colleagues have shown that muscular contractions are involved in reducing the epithelia of the wound site and potentially favoring the wound-healing process [62]. In addition, a stereotyped tissue dynamics that may reflect the above-mentioned observation of MyHc1::mCherry positive cells retracting from the amputation site, supports the idea that muscle contractions play also a crucial role during various phases of the regenerative process [59]. A recent study has shown that the retractor-muscle containing mesenteries are fundamental in inducing regeneration in nematostella [48]. Based on data from planarian [63] and mammalian myoepithelial cells [64], one could thus speculate that the epitheliomuscular cells that form the retractor muscle are also involved in the regeneration process via contraction-independent biochemical signals. However, additional work is required to further support those evidences and to determine the cellular and molecular mechanisms involved.

## 4. Platyhelminthes: The Freshwater Planarian

Since the beginning of the 21st century, the freshwater, free-living, flatworm planarian, has become a leading model for the study of development and regeneration mechanisms [65]. As a model organism, it possesses a set of clear advantages. 1. Reductionism: although its relative simplicity, planarians exhibits much of the “complexity” of vertebrate systems, including a well-differentiated nervous system, simple eyes, central brain, triploblastic organization, and bilateral symmetry. 2. Planarians are inexpensive and very easy to rear and maintain in the lab, therefore ideal for primary high-throughput screening processes [66]. 3. Planarians are molecularly-tractable model organisms, easily manipulated by RNAi interference [67] and their thin and somewhat transparent body allows whole-mount in situ hybridization in an intact worm [68].

However, there is no doubt that the most astounding feature is its regeneration capability. Planarians are considered as the “Masters of Regeneration”. Adult pluripotent stem cells that are called neoblasts and are the only proliferating cells, account for 25–30% of all cells distributed in the planarian body, and give them remarkable regenerative abilities. Whole worms can regenerate from only a small proportion of the adult worm, within 1–2 weeks. Consequently, full results from regeneration experiments are revealed in a relatively short time. For a broad review of planarian as a model system for regeneration see Ivankovic et al. [69].

### 4.1. Muscle Types

The planarian’s muscle cells combine features common to both skeletal and smooth muscles [63]. The planarian contains two main muscular systems, somatic and pharyngeal, that differ in their myosin heavy-chain (MHC) muscle isoforms along with their function and location possibly due to their different biological functions [63]. Without a supportive skeleton, the maintenance of body shape, posture, movements, and defense (strength for their soft bodies) depends on the somatic muscular system. Locomotion is mainly executed by ciliary gliding. The muscular body wall, organized beneath the epithelium, is arranged in a grid work of 4 layers of fibers lying in different orientations and linked to an extracellular network of filaments associated with the body’s organs [63]. Moreover, recent works [70,71,72] revealed that in planarian, the muscles also provide patterning signals essential to regeneration and guidance of tissue turnover and regrowth after injury. Interestingly, this resembles what has been suggested to be a function of the connective tissues in vertebrates [73]. The somatic muscular system provides regeneration guidance through the expression of position control genes (PCGs) differing over time, body region, and types of genes expressed.

In addition to the somatic muscular system, planarians possess a separate pharyngeal musculature system. Planarians have an incomplete digestive system with pharynx (proboscis and anus) connected to the intestine duct by its anterior thus providing a single opening that functions as both, anus and mouth. The pharynx is composed of a muscular tube and demonstrates repertoires of movement capabilities. It is extruded from the body center during feeding and can direct itself toward the food by bending and stretching till it reaches the food; it thus swallows the food and transfers it to the intestine by peristaltic movements. The pharynx does not contain neoblasts [74] and therefore is incapable to regenerate the rest of the worm when amputated. However, a worm losing its pharynx can regenerate it in a few days [75]. The pharynx can thus serve as a module of organ regeneration where stem cells differentiate into distinct cell types to form an organ that integrates within the rest of the body [76].

### 4.2. Muscle Regeneration and Homeostasis

Upon regeneration, planarian muscle cells, as all other tissue components, arise from the large reservoir of the existent neoblasts population that migrate to the wound area and start proliferate, thus creating a blastema where they differentiate to form the missing body parts. Irradiation protocols applied to the whole body or to specific areas allows neoblasts ablation [77]. Further transplantation of a single pluripotent neoblast can restore regenerative ability and the whole process can be monitored from scratch [78]. Therefore, planarian is an ideal model for deciphering the mystery of stem cell differentiation [79], allowing experimental approaches that are unavailable in any other model organism. Research on planarian muscle regeneration is still limited but provides some interesting perspectives.

One other unique feature of the planarian model (e.g., *Dugesia japonica* and *Schmidtea mediterranea*) is the ability to shift from growing (up to few centimeters) to de-growing (down to few millimeters) by food deprivation and vice versa. The process depends on the balance between cell proliferation and cell death and by keeping stable body shape and proportions through constant remodeling mechanisms [80]. Therefore, it is a perfect model system for the study of tissue homeostasis (for a broad overview of the subject see [81]).

In spite of their exceptional features and their growing popularity as a model for basic research on regeneration, planarians are not yet considered as a conventional organism for studying human pathologies and diseases, maybe it is time to rethink [82].

## 5. Mollusks: The Cephalopods

Cephalopods represent one the main and most evolved mollusk class. They are the most intelligent, mobile, and the largest of all mollusks and include very diverse species such as squid, octopus, cuttlefish and the chambered nautilus.

Regeneration is a frequent event occurring during cephalopods’ lifetime. Wild animals often lose body parts such as portions of arms and fins and as a consequence, it is common to find signs of traumatic events on their bodies [83,84]. These events can dramatically impair their capacity to swim, capture, and manipulate preys [85], and therefore they can seriously impact their survival in the natural environment. Indeed, cephalopods can regenerate their cornea, peripheral nerves, and body limb (arms and tentacles) [86]. Cephalopod limbs are complex organs composed of a tightly packed three-dimensional array of muscle fibers controlled by a sophisticated peripheral nervous system (PNS). The arm PNS is composed of three distinct parts: the arm nerve cord (composed of axial nerve cords and the ganglionic core), the sucker ganglia, and the intramuscular nerves. This assembly allows the transmission of a large amount of sensory and motor information to and from the brain [87,88,89,90]. All of these structures are fully and functionally recovered during regeneration.

### 5.1. Muscle Types

Similar to vertebrates, muscle cells in cephalopods can be found in a variety of different organs that differ dramatically in structure and function. Indeed, muscle cells are in the mantle and appendages (arms and tentacles) but also in eyes, hearts, viscera and chromatophores. Such diversity is paralleled by specific adaptations in muscular organization and physiology.

The majority of the musculature of arms and tentacles is composed by uninucleate transverse or obliquely striated muscle fibers with shared morphological and physiological characteristics. When oblique striation is present, this pattern is uniform and continuous among adjacent cells. Generally, these muscle cells do not exceed 8–20 µm in diameter and 0.8–1 mm in length. The nucleus is in the central portion of the cell whose transverse section is usually round or polygonal, with a mitochondria-rich core and a contractile apparatus in the cortical zone. The contractile apparatus lies along the main axis of the fiber and is organized in sarcomeres with identifiable acto-myosin striations. Cephalopod muscle actin and myosin heavy chain, show strong sequence identity to other invertebrates and vertebrate gene orthologs suggesting a similar contraction mechanism [91,92]. On the contrary, regulatory proteins are very cephalopod-specific [93] suggesting that specific control kinetics and cross-bridge cycle regulation might be developed in cephalopods (for a review see [94,95]). Different from typical skeletal muscles, cephalopod arm muscle cells do not possess a proper T-tubules system, but smaller sarcoplasmic structures named “terminal cisternae” that take contacts with plasma membrane invaginations thus forming “dyads” at the level of the Z-disks. In contrast to the muscle cells of other invertebrates, they are isopotential, and thus each synaptic input can control the membrane potential of the entire muscle cell (for a review see [94]).

Among cephalopods, muscle cells can differ in their activation properties. As an example, in octopus arm, muscle action potentials rely on Ca^2+^ spikes [96,97] generating a massive entrance of Ca^2+^ that activates a calcium-induced calcium release (CICR) process from the internal stores [98]. An intriguing analogy here can be found with vertebrate cardiac muscle cells that represent an important target of regeneration medicine [99,100].

In contrast to the octopus arm muscles, transverse muscles of squid tentacles show ‘graded’ Na^+^ based action potentials different from the typical ‘all or nothing’ action potentials of squid giant axon or vertebrate muscle fibers. Interestingly, the transverse muscle of the squid arms lacks Na^+^-based action potentials [101]. All the above-mentioned characteristics co-evolved with the complex brain to body adaptation and limb specialization [102,103,104] whose integrity is essential to the animal survival.

Several myogenic genes have been identified in some (but not all) cephalopod species. As an example myoblast-specific Myf5 and MyoD proteins have been identified in *Sepia officinalis* tentacles during late stage development and NK4 is found to be involved in cephalopod striated muscle formation just as in vertebrate cardiac cells [94,105,106]. In addition, an hh-homolog signaling molecule and its receptor Patched (Ptc) have been found to be expressed during myoblast differentiation in *Sepia officinalis* [107].

### 5.2. Muscle Regeneration

Cephalopod mollusks are a powerful model of limb regeneration due to their similarities in early arm development to vertebrate models and their fast and efficient regenerative abilities (for a review see [94]) and, among regeneration studies of other body parts, rather ample literature is currently available on the regeneration of their limbs (for a review see [84]). However, very little is known about the molecular pathways controlling the regenerative process.

Hereafter, we will employ the octopus arm as a template to describe the step of a regeneration process. Morphologically, a sequence of events can be identified during arm regeneration: (1) wound healing; (2) formation of a knob at the stump tip; (3) elongation of the knob and formation of a hook-like structure; and (4) elongation of the regenerating arm till complete restoration of a functional structure [85,94,108,109].

At early steps of regeneration, a mixture of extracellular matrix (ECM), vesicles, and mucus are present at the plug region, and only subsequently the connective tissue is deposited by fibrocytes migrating to this region [109]. The presence of ECM and connective fibers might be relevant for the correct reorganization of the regenerating structure, a role that has been also suggested in octopus pallial nerve regeneration [84]. Cephalopods might have evolved fine mechanisms of regulating ECM composition and organization during regeneration that favor the tissue competency to regrow. Interestingly, similar fibrillary elements are the ones limiting vertebrates skeletal muscle regeneration as their accumulation at the injury site negatively interferes with regeneration and drives instead scarring and fibrosis of the tissues [94].

At a cellular level, cells composing the stump are first characterized by a layer of undifferentiated cells together with diffuse vascular components forming a typical blastemal region. This structure then disappears, and cells start differentiating [110]. Cell proliferation remains active throughout the entire regeneration process, but while at an early step is primarily localized at the blastema, at later stages it is present within differentiating tissues such as the axial nerve cord and the musculature.

Unfortunately, no study reported so far could reveal the molecular identity of muscle cell precursor during regeneration. It has been speculated that new muscles and nerve cells can originate from dedifferentiated cells of the same type (for a review see [84]) but due to the lack of species-specific molecular markers, we are currently not able to assess the existence of pluripotent vs. lineage-committed progenitor cells, as well as vertebrate satellite-like cells associated with adult muscles. From a mechanicistic viewpoint it has been shown that after an arm lesion, muscles close to the injury site degenerate fast, and large cells containing little protoplasm and a large nucleus appear within the same area. These cells are supposed to be sarcoblasts that later migrate to the most distal part of the wound and undergo active proliferation. Sarcoblasts will then differentiate into the arm and sucker muscle fibers in different time intervals [108]. This process is possibly paralleled by the recovery of the arm functional capacity.

Few data are available on the molecular pathways underlying muscle formation during regeneration in cephalopods. It is known that cephalopods muscle development rely on MRFs, however, still, no data are available on their expression during muscle regeneration in octopus. Several studies suggested that acetylcholinesterase (AChE), a conserved molecule between vertebrates and invertebrates, may orchestrate the formation of the octopus arm during regeneration [110,111] similarly to what happens in regeneration phenomena occurring in other animal phyla such as Platyhelminthes, Mollusca, Arthropoda, and even Chordata (for a review see [94]).

## 6. Nematodes: The *Caenorabditis elegans* Model

Nematodes are one of the most diverse animal phyla. They occupy a large variety of environments, and many species are parasitic. Nematodes are relatively small animals (~1 mm long adults), and, given their size, a heart and a closed circulatory system are not required.

*C. elegans* has been employed as a model to study extrinsic and intrinsic factors crucial for axon regeneration and wound healing. In particular it have disclosed important aspects of the mechanisms of wound healing and cellular plasticity, axon regeneration and transdifferentiation in vivo [112].

### Muscle Type and Homeostasis

The majority of muscles of the animal body wall are used for the animal’s locomotion [113]. *C. elegans* body wall muscle cells are spindle-shaped mononuclear cells with multiple sarcomeres per cell [114]. Muscle cells are obliquely striated and form body-wall muscles running along the length of the body underneath the epidermis [115,116]. Unlike most other animals, their innervation is unusual in that the nerves do not branch out into the muscles but the muscle cells send extensions (muscle arms) to the nerve cord to receive *en passant* synapses from the motor neurons [117,118].

Embryonic development of body wall muscle is controlled by maternally expressed genes initially, but then there is a switch to control by zygotically expressed genes. Several molecular players (e.g., Wnt/Mitogen-Activated Protein (MAP) kinase signaling, Myogenic regulatory factors (MRFs) as many others) act during muscle development and differentiation. For a detailed description please refer to [119,120].

*C. elegans* muscles lack satellite cells (muscle stem cells) and therefore muscles cannot regenerate. Adult worms only carry post-mitotic body wall muscles [119]. It is interesting to notice that, although lacking an open circulatory system, proteins and structures composing the body wall muscles manifest a high homology with that of human heart muscle. In addition, many molecules involved in sarcomere assembly and maintenance are in common with other animals. A dystrophin ortholog, dys-1 gene, has been identified in *C. elegans* with a key role in the sarcomere structural regulation [121]. The mechanism of assembly of sarcomeres into functional muscles have been extensively investigated in *C. elegans* within the context of repair following activity-induced muscle stress and muscle degeneration [117].

For the reasons listed above, *C. elegans* has become a model study for muscle diseases such as Duchenne’s Muscular Dystrophy (DMD) [122] and cardiomyopathies [113]. A more explanatory and detailed list of advantages and disadvantages of this animal as a model of human heart pathologies can be found in [113].

Interestingly, the lack of regeneration capacity of *C. elegans* muscles has been key to the use of this animal as a model of DMD. Indeed, as *C. elegans* adult muscle cells are mono-nucleated and post-mitotic, they can be individually tracked during the process of muscle degeneration and do not undergo fibrosis and chronic inflammation, processes that are common in vertebrate models [121].

## 7. Artropods: The Insect *Drosophila melanogaster*

Insects have a reduced lifespan and events related to degeneration/regeneration processes following physical, pathological or aging damage are less frequent. Hence, the establishment of a real physiological regenerative mechanism have been under a lower evolutionary pressure. However insects manifest adult muscle hypertrophy, which can be viewed as a degeneration/regeneration-like process, in response to particular hormones as well as to environmental factors, population density, food availability, or mating [123,124].

*Drosophila melanogaster* is a model organism in which genetic and molecular techniques, coupled with physiological and structural approaches, have been used to unravel specific issues of invertebrates and vertebrates muscle biology, including regeneration processes.

### Muscle Type and Homeostasis

In *D. melanogaster* larvae and adults, three types of muscles can be recognized: (1) Tubular muscles, including most of the adult skeletal muscles. They are striated with a centrally located nucleus and are synchronous muscles because each nerve stimulation evokes calcium release from internal stores which triggers a mechanical contraction of the muscle similarly to vertebrate skeletal muscles [125]. (2) Adult indirect flight muscles, or “fibrillar” muscles. In these muscles individual myofibrils can be identified by light microscopy; they are striated and asynchronous muscles as their mechanical response is activated both by calcium following nerve impulse and by stretch-activation due to the elastic recoil of thorax cuticle [125,126]. (3) Supercontractile striated visceral and heart muscles, and larval body wall muscles. Supercontractile muscles are called “supercontractile” because they can contract to a length well below 50% of their resting length [127,128,129,130,131]. They contract in response to caffeine also in the absence of external calcium, showing that a functional store of calcium is present in the sarcoplasmic reticulum and that it is sufficient for muscle contraction [132]. Interestingly, a similar activation property has been also found in the octopus arm muscles [98].

Larval and adult muscle cells and fibers derive from progenitor cells of the embryonic mesoderm. Signaling crosstalk between ectoderm and mesoderm (for instance Decantaplegic and Wingless) and gene (e.g., *twist*, *even-skipped,* and *floppy-paired*) dynamic temporal expression, regulate the muscle cell fate of these cells [133,134]. Cardiac and visceral muscle cell progenitors are formed from these generic muscle cell progenitors by their compartmentalization in segmental regions with low Twist high Even-skipped domains. High Twist high Sloppy-paired domains are, on the other hand, a key point for the development of somatic cell progenitors [134,135]. Both these muscle cell progenitors undergo then asymmetric cell divisions, which generate low Twist cells that fuse to form embryonic myoblasts and subsequently embryonic muscles. Some, but not all, asymmetric division give rise also to a single founder cell and to an adult muscle precursor (AMP), an adult muscle stem cell that remains in quiescence.

Larval muscles degenerate throughout metamorphosis. In some cases (indirect flight muscles in the thorax) larval muscles are utilized as templates for the formation of adult muscles. In other cases, peripheral nerve fibers and the space between larval muscle fibers drive adult muscle fibers development and differentiation. Adult muscle fibers origin from AMP precursors which proliferate, differentiate, and fuse to form myotubes and then adult fibers. This process was deeply studied in indirect flight muscles that are the main power source for flight. From these studies, however, it was found that only a small number of AMPs descendent stem cells remain associated with the adult differentiated indirect flight muscle fibers. These cells resemble mammalian satellite cells which are associated with the adult skeletal muscle fibers and retain the competence to proliferate and differentiate in myoblasts and then adult myofibers when stimulated (for example following skeletal muscle fiber degeneration). It is interesting to note also that these “fly satellite cells” undergo a proliferation/differentiation program leading to the generation of myoblasts which fuse with a damaged indirect flight muscle fiber. This process, similarly to what was observed in vertebrate satellite cells of skeletal muscle fibers, points to repair the damaged fibers, and it is activated by Notch-Delta signaling [136]. In the absence of tissue damage, satellite cells are maintained, not differentiated, and “quiescent” probably by the transcription factor Zhf1 [137].

Other authors claim that there are probably no “satellite cells” in adult flies’ muscles. Indeed, almost all of these studies were investigating the indirect flight muscles (IFMs) that are considered the most similar to mammalian skeletal muscles. In these, regeneration processes are triggered by damage consequent to physical or pathological injuries as well as damages related to aging. Considering fly lifespan, these events are less frequent and therefore they should have exerted a minor pressure from the evolutionary point of view to establish a real regenerative mechanism in adult muscles. Moreover, a regeneration-like process is considered for adult skeletal muscles as regarded as muscle hypertrophy. Again, in flies, differently from other invertebrates, the small dimension of IFMs could have been a factor against a “pressure” from an evolutionary perspective.

## 8. Echinoderms: A Compendium of Regeneration Strategies

Echinodermata is a phylum consisting of radially symmetrical marine animals. All larval and adult echinoderms exhibit high regenerative capacities of entire lost parts following predation or traumatic events [138]. Echinoderms manifest all the regenerative strategies identified in other animals, such as epimorphosis and morphallaxis, and have an impressive high genetic homology with Chordates. They can show epimorphic processes, by which a blastemal is formed through active proliferation of migratory undifferentiated cells. They can also show morphallaxis, where cells derive from differentiation, transdifferentiation, or migration of existing tissues. Most classical and bio-molecular tools currently available have been successfully employed in this animal species giving rise to a large body of literature on Echinoderms regeneration from molecular, cellular, and tissue level. These features make them interesting models in translational research [139].

### 8.1. Echinoderm Muscles

Movements in echinoderms are assured by a muscular and a water vascular system; two main muscular systems, the visceral and the somatic, are present. Similar to what happens in nematodes and amphioxus, echinoderm visceral muscles may extend cytoplasmic prolongations towards the nerve fibers that they make contact with. Echinoderm muscles retain some epithelial features. Indeed, the epithelial cell of coelom can give rise to peritoneocytes, myoepithelial cells, and myocytes through successive stages of specialization. Despite differences in anatomical location, echinoderm muscles share a similar structure. They are made up of numerous contractile bundles and each of them is composed of several myocytes containing myofilaments of variable thickness [140].

Two main types of muscle fibers can be identified, the first (and most common) in which individual bundles are composed of myocytes of fusiform shape and resemble vertebrate smooth muscle fibers. These fibers are embedded in the extracellular matrix of connective tissue composed of a network of thick striated (collagenous) and thin unstriated fibers and an amorphous component; it also comprises fibroblasts, nerve cells, and different coelomocytes (the immune effector cells of sea urchins). A second muscle type, typical of crinoid arms, consists of obliquely striated fibers with each muscle bundle composed of 8–20 myocytes and surrounded by a basal lamina.

### 8.2. Echinoderm Muscle Regeneration

Several signaling pathways are involved in regeneration. These include the bone morphogenetic protein/transforming growth factor (BMP/TGFB)-signaling pathway, the homeobox (HOX) signaling pathway and the Ependymin pathway. Nonetheless, it is not directly possible to associate these pathways with the specific process of muscle regeneration. To provide this information, it would be necessary to screen for genes expressed by muscle cells such as cytoskeletal genes, actin, and myosin genes. Interestingly, their expressions are known to be modulated during different stages of the regeneration process [141,142,143].

Upon injury, echinoderm muscles undergo processes of de-differentiation and myogenesis. In the wound region damaged myocytes degenerate and muscle bundles disintegrate. De-differentiation of the coelomic epithelial cells represents an early regeneration event occurring already during wound healing and continuing at different rates during the regenerative period.

These cells dedifferentiate and start migrating toward the region occupied by the injured muscle, here they form clusters of muscle bundle rudiments. Then, they increase in number and start developing the contractile filaments of future muscle cells. The process of myogenesis goes on in parallel with many other regenerative events and brings to the restoration of a functional muscular tissue [142,144].

In conclusion, echinoderms represent an interesting model with a high potential for muscle regeneration studies (for extended reviews see also [138,142,144]). Their close phylogenetic relation to vertebrates makes them attractive models to determine what cellular and molecular processes are required for successful muscle regeneration to occur.

## 9. Cephalochordates, the Basal Chordates: Amphioxus

The cephalochordate amphioxus is the sister group of the tunicate–vertebrate clade [145] (Figure 1) and represents a new emerging model for studies on cell and tissue regeneration and in particular for muscle regeneration. Its phylogenetic position also offers insights into the evolution of regenerative capacity in vertebrates.

As demonstrated in other organisms, amphioxus regeneration can vary among body parts and several variables affect the speed of healing such as species, animal age, and body size [146,147,148,149]. In amphioxus, two districts show the highest regenerative capacity: the oral cirri, skeletal structures surrounding the mouth, and the post-anal tail.

### 9.1. Structure of Amphioxus Muscles

The adult amphioxus possesses almost exclusively striated muscles, the most prominent of which are the segmental axial muscles providing force for burrowing and swimming, the notochord and the pterygial muscle. The axial musculature is composed of the myomeres thatare segmentally repeated in dozens of pairs throughout the entire length of the body. Myomeres are composed of flattened striated muscle cells 0.8 μm thick [150], 100 μm wide and at least 500 μm long. They are similar to vertebrate skeletal muscle cells in banding and in the arrangement of the myofilaments [151]. Moreover, the sarcoplasmic reticulum, although not arranged to form the typical T-tubule system, is present and, as in vertebrate striated muscles, might serve as calcium storage [152]. Despite these similarities, amphioxus axial muscle cells are mononucleated, in contrast to the fused myofibers of vertebrate skeletal muscles. The notochord, a modified muscle structure extending from the tail to the most anterior tip of the rostrum, consists of a row of coin-shaped striated muscle cells plus other non-muscle cells known as Müller cells. The pterygial muscle is constituted by striated fibers [153] responsible for the contraction of the branchial cavity, which results in gamete emission from the atriopore.

### 9.2. Muscle Regeneration in the Amphioxus Tail

**Figure 3:**
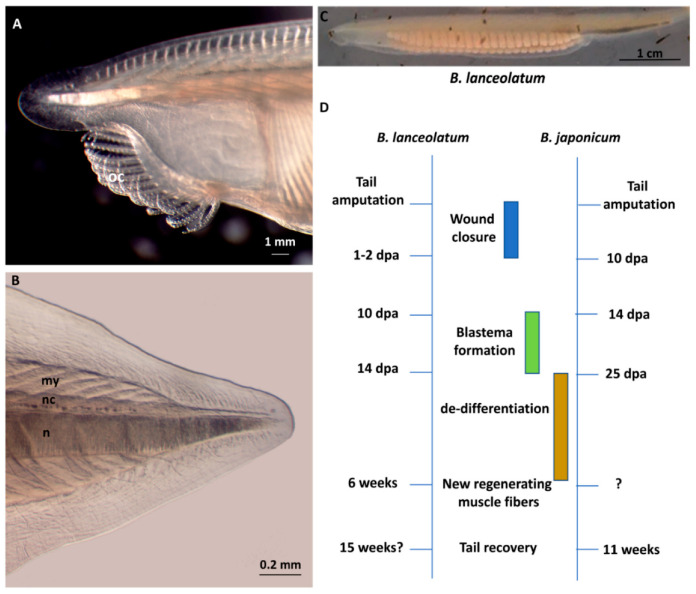
Timing and principal steps of regeneration in *B. lanceolatum* and *B. japonicum*. (**A**) Enlargement of the most anterior end of a *Branchiostoma lanceolatum* adult showing the oral cirri (oc). (**B**) Post-anal tail of the same animal. my, myomeres; nc, nerve cord; n, notochord. (**C**) *B. lanceolatum* individual collected in Banyuls-sur-Mer, France. (**D**) Schematic overview of the series of events occurring during tail regeneration in the *B. lanceolatum* and *B. japonicum*. dpa, days post-amputation.

Due to its ability to regenerate skin, nerve cord, and muscles after amputation, the post-anal tail is the most studied system for understanding regeneration in amphioxus (see Figure 3 for timing and principal steps of regeneration in *B. lanceolatum* and *B. japonicum*). Indeed, the tail contains the two most prominent striated muscle structures: the myomeres and the notochord.

While little data are available on the role of myogenic factors during tail regeneration in amphioxus, much more is known on muscle development formation in the embryo. Like vertebrates, amphioxus axial muscles derive from the myotomal portion of the somites. Here, the Six1/2-Pax3/7 myogenic program is activated [154] and regulates the differential expression of members of the myogenic regulatory factors (MRFs) gene family, which underwent independent expansion in the amphioxus lineage [155]. Amphioxus somitic mesoderm, which extends for the whole length of the body, is divided into three portions, each induced by a unique combination of transcription factors [154]. The most anterior somites depend on fibroblast growth factor (FGF) signaling [156]. The signals regulating the other two populations are yet to be identified but it has been shown that the most posterior somites, arising from the tail bud as the embryo elongates, do not require FGF nor retinoic acid signaling [156] and that Notch is required for correct separation of contiguous somites [157].

From a mechanistic viewpoint, Somorjai and coworkers [146,147] described a blastema-like structure in the amputated tail of *B. lanceolatum*, with proliferating cells from notochord, myotomes and nerve cord positive for phospho-histone H3. Subsequently, Liang and coworkers [148] confirmed cell proliferation in blastemal cells of the regenerating tail of *B. japonicum* by Bromodeoxyuridine (BrdU) labeling. Conversely, Kaneto and Wada [149] identified in the amputated oral cirri of *Branchiostoma belcheri* a large number of mesenchymal cells able to reform the skeleton, but most likely without the influence of a proliferative cell population, as phospho-histone H3 was not detected. Thus, oral cirri seem to undergo tissue remodeling by morphallaxis, whereas the tail can respond to amputation injury by epimorphic regeneration. In addition, de-differentiation of existing structures but not trans-differentiation or lineage reprogramming seems to occur during amphioxus tail regeneration, as seen in amphibians [146,147].

## 10. Tunicates: The Sister Group of Vertebrates

**Figure 4:**
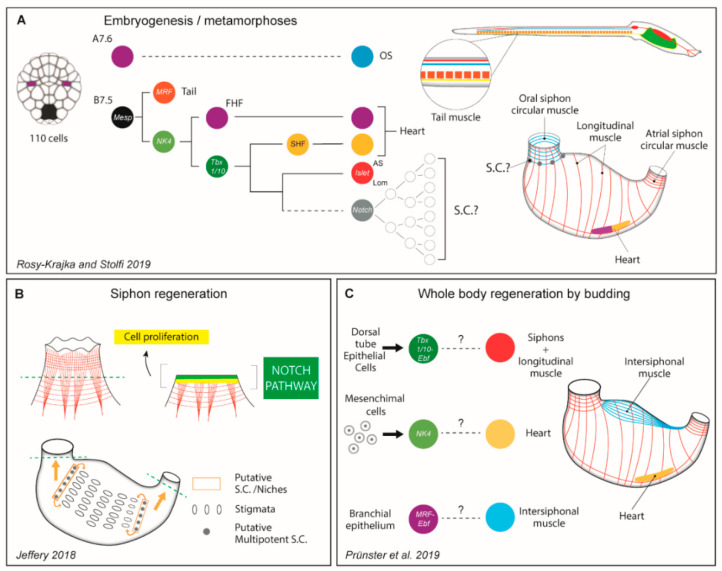
Ciona embryogenesis and regeneration. (**A**) Simplified scheme of muscle development during ciona embryogenesis and metamorphoses. (modified from [163]). (**B**) Scheme of oral siphon regeneration in ciona (modified from [164]). (**C**) Simplified scheme of myogenesis during budding in *Botryllus schlosseri*. FHF: first heart field; SHF: second heart field; OS: oral siphon; AS: atrial siphon; LoM: longitudinal muscles; SC: stem cells.

Tunicates (Phylum Chordata) encompass a large group of ubiquitous and diverse animals that occupy a wide variety of marine habitats and ecosystems around the world [158]. Despite their appearance, these animals are the sister group of vertebrates, with whom they share a common ancestor [159]. Most of the tunicate species have a biphasic life cycle, with a swimming larva, with the chordate synapomorphies, and either a benthic (like the group of ascidians) or a planktonic (in the order thaliaceans) post-metamorphic phase. During this phase, the chordate features are lost and the larva turns into a filter feeder sac-like body structure with two tubular openings, known as inhalant and exhalant siphons (Figure 4). While solitary tunicates reproduce strictly sexually and have limited regenerative capabilities, colonial species can also reproduce asexually and regenerate an entire body via diverse modes of budding, also referred to as non-embryonic development. The result is often a colony of connected, genetically identical zooids [160,161]. Budding can be part of the life cycle, which accounts for colony growth, replication and reproduction, or regeneration, i.e., passive forms triggered by injury [162].

### 10.1. Myogenesis during Embryogenesis and Metamorphoses

The typical tunicate larva carries in the tail two bands of mononucleated muscle cells, one on each side of the notochord. The myofibrils of each cell are connected through intercellular junctions, similarly to the vertebrate cardiomyocytes. Each row of cells behaves as a syncytium, allowing the swimming movement. The myocytes are arranged in a striated manner and express myosin heavy chain. After metamorphosis, the sarcomere organized musculature gets reabsorbed along with the tail, and the musculature of the formed zooid generally consists of longitudinal and circular multinucleated fibers that run throughout the mantel along the body and around the two siphons (Figure 4). While ultrastructural studies show unstriated morphology, the adult body musculature seems to have intermediate characteristics between the vertebrate smooth and striated muscles [165]. For instance, the post-metamorphic musculature uses a troponin-tropomyosin complex similar to vertebrate striated muscles, their myocytes are specified via MRFs that generally controls vertebrate skeletal muscle development, and lacks on smooth muscle specification [165,166]. Post metamorphic zooids also develop a tubular heart, which consists of a single chamber formed from two epithelial monolayers. The pericardium is a non-contractile epithelium, whereas the myocardium is a single layer epithelium of mononucleated striated cardiomyocytes.

Most of the information on the molecular bases of muscle cell specification and differentiation comes from a copious amount of studies focused on few solitary model species namely *Ciona intestinalis*, *Halocynthia roretzi,* and Molgula, and have been recently summarized in a comprehensive review by Razy-Krajka and Stolfi (2019) [163]. Briefly, during embryogenesis, the maternal deposition of a zinc finger family member (Zic-r.ca) and the activation of a T-box transcription factor is necessary to induce the development of tail muscles. At the stage of 110-cells, a couple of blastomeres, the B7.5, acquire a cardiopharyngeal mesodermal fate and give rise to both heart and part of the adult body musculature, specifically the exhalant siphon and the longitudinal muscles (Figure 4). The gene regulatory network that governs this cell lineage specification is highly conserved between tunicates and vertebrates cardiopharyngeal myogenesis [167]. Another couple of blastomeres (the A7.6) follow a different fate but are partially regulated by the same transcription factors involved in the cardiopharyngeal specification [107] and give rise to the muscles of the inhalant siphon (Figure 4A).

### 10.2. Myogenesis during Budding and Regeneration

Many solitary ascidians can repair and regenerate efficiently both the exhalant and inhalant siphons, including the associated muscle fibers [168]. Interestingly, during its progressive differentiation, the B7.5 cell lineage gives rise also to a population of cells that do not express the MRF, but seems to maintain an undifferentiated state and settle around the exhalant siphon [169,170]. These muscle precursors maintain a stem cell-like state via a Notch-mediated lateral inhibition, a mechanism that has been also reported in drosophila and vertebrates to control muscle differentiation [170]. In addition, the A7.6 is multi-lineage, but it is not clear if such multipotency is retained in the fully developed adult. So far, the link between the B7.5 and A7.6 myogenic lineage and the siphon muscle regeneration has not yet been explored. Recently, Jeffery (2018) suggested that, in *Ciona intestinalis*, the siphons repair and regeneration are triggered by the mobilization of multipotent progenitors that migrates from niches located in the branchial sac rather than around the very same siphon [164]. The very same stem cells are also responsible for the regeneration of the central nervous system. In addition, the nature and the dynamics of these stem cells have not been yet described (Figure 4B). While there are no recent studies on *bona fide* heart regeneration in tunicates, growth regions have been reported in the ciona myocardium [167,171]. In these area, clusters of proliferating undifferentiated cells start to accumulate myofilaments and eventually mature into cardiomyocytes. The nature of these precursors, i.e., transdifferentiating cells or cardiac stem cells, remains to be studied.

Although way less studied, the embryogenesis of most colonial tunicates seems to occur in the same way than the solitary ones [172,173] and, at least in the model *Botryllus schlosseri* the myogenic regulative modules and mechanisms appears to be conserved [174]. The blastozooid, the adult produced by non-embryonic development, generally has a bauplan and a muscle architecture that is comparable to the oozooids, i.e., the individuals formed by embryonic development [165,174]. However, budding bypass fertilization, embryogenesis, larval stage, and metamorphosis [24,160,171]. Contrary to their embryonic development, which displays a remarkable level of conservation among almost all the tunicate orders, non-embryonic development encompasses a clade-specific assortment of cells, tissues, and ontogenesis, all displaying different degrees of interaction between epithelial and mesenchymal cells [161].

In *Botryllus schlosseri*, the blastozooid musculature is formed de novo during morphogenesis by partially co-opting and re-wiring the embryonic cardiopharyngeal regulatory network [174]. The body muscle fate seems to be regulated by a kernel of genes expressed in progenitor cells located in a transitory structure, the dorsal tube, which has also neurogenic potential [173]. Instead, the hierarchy of the expression of specific cardiomyogenic transcription factors suggests that the heart is specified by different mesenchymal precursors, located in another domain of the developing bud. Therefore, the reshuffling of the embryonic cardiopharyngeal regulatory modules is also linked with uncoupling of the body muscle and heart muscle precursors [174]. It does remain unclear if these populations of precursors are renewed every budding cycle or persist and pass over asexual generations, or if the same precursor is responsible for the myogenesis during other forms of partial or total regeneration [175] (Figure 4C).

In the other two ascidian species, *Botrylloides leachii* and *Perophora viridis,* a population of adult pluripotent stem cells circulating in the hemolymph seems to be responsible of the regeneration of the whole body, including the entire musculature [176,177]. As for solitary species, the study of these cell populations is still in its infancy.

## 11. Vertebrates: The Zebrafish

**Figure 5:**
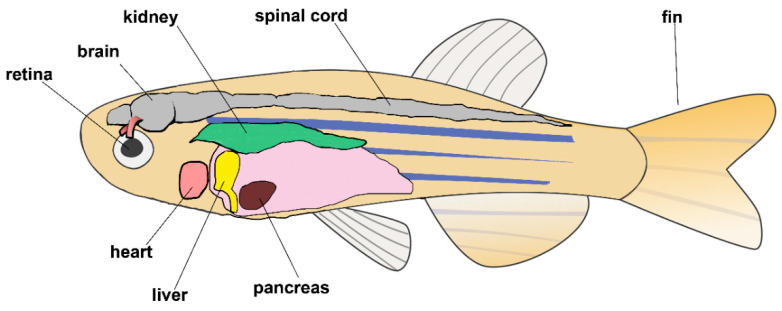
Regeneration in zebrafish. Schematic drawing of a zebrafish adult showing organs used for regeneration studies.

The zebrafish (*Danio rerio*) is one of the most widely used vertebrate model for regeneration studies. Zebrafish are capable of regenerating many of their organs and tissues, including heart, central nervous system, retina, lateral line hair cells, caudal fin, kidney, pancreas, liver, and skeletal muscle (reviewed in [178,179,180]; Figure 5).

### 11.1. Zebrafish Skeletal Muscle Regeneration

Zebrafish trunk is composed of spatially separated slow and fast muscle fibers, and slow myofibers are embryonically mononucleated (reviewed in [181,182]). The trunk muscles are arranged in repeated chevron-shaped segments, along the head-to-tail axis. The thin partition between each pair of adjacent somites is named ‘vertical myoseptum’ and the one between the dorsal and ventral halves is named ‘horizontal myoseptum’. The myosepta are anchoring structures for muscle fibers, enabling force transmission [183]. Two main Pax7-positive muscle stem cell populations were characterized in zebrafish. The first population is formed after somitogenesis at the external surface of the myotome, the external cell layer (ECL), expresses Pax3 and Pax7, and contributes to muscle growth throughout the zebrafish lifespan by secondary myogenesis [184]. The second population is functionally equivalent to the amniote satellite-cell population, scattered between myofibers throughout most of the myotome and serves as a source of new muscle fibers during adult zebrafish muscle repair and regeneration. These satellite-cells are mainly enriched in slow muscle near the myosepta, have dense heterochromatin and express Pax7, Pax3, and Met. In response to muscle injury, they divide asymmetrically to form two distinct cell pools: proliferative cells that fuse to form de novo myofibers or repair damaged muscle fibers, and proliferative cells that self-renew to ensure the preservation of a satellite stem cell pool [180,185]. Zebrafish skeletal muscle is a heterogeneous tissue, composed of slow, fast, and intermediate myofibers. However, at variance with mammals’ intermixed muscle bundles, zebrafish trunk is composed of spatially separated slow and fast muscle fibers, and slow myofibers are embryonically mononucleated (reviewed in [181,182]). The trunk muscles are arranged in repeated chevron-shaped segments, along the head-to-tail axis. The thin partition between each pair of adjacent somites is named ‘vertical myoseptum’ and the one between the dorsal and ventral halves is named ‘horizontal myoseptum’. The myosepta are anchoring structures for muscle fibers, enabling force transmission [183].

Myogenesis events are fundamentally common to all vertebrates. The myogenic regulatory factors (MRFs) that direct myogenic lineage development and muscle differentiation (i.e., Myf5, MyoD, Myogenin, Mrf4) are highly conserved in fish and mammals (reviewed in [178,179,180,186]). Two main Pax7-positive muscle stem cell populations were characterized in zebrafish. The first population, the external cell layer (ECL), is formed after somitogenesis at the external surface of the myotome. It expresses Pax3 and Pax7 and contributes to hyperplastic muscle growth throughout the zebrafish lifespan by secondary myogenesis [184]. The second population is functionally equivalent to the amniote satellite-cell population, scattered between myofibers throughout most of the myotome and serves as a source of new muscle fibers during adult zebrafish muscle repair and regeneration. These satellite-cells are mainly enriched in slow muscle near the myosepta, have dense heterochromatin and express Pax7, Pax3, and Met. In response to muscle injury, they divide asymmetrically to form two distinct cell pools: proliferative cells that fuse to form de novo myofibers or repair damaged muscle fibers, and proliferative cells that self-renew to ensure the preservation of a satellite stem cell pool [180,185].

### 11.2. Zebrafish Heart Regeneration

The zebrafish heart is simpler than the mammalian heart and is composed of a single atrium and a single ventricle. Blood exits the heart through the *bulbous arteriosus*, an elastic, non-contractile chamber composed of smooth muscle. The wall of the zebrafish ventricle is lined by the epicardium, an outer mesothelial lining, and an inner endothelial lining, the endocardium. The wall is composed mainly by muscle cells, it is vascularized and innervated, and contain also fibroblast and several other type of cells [187].

Poss et al., in 2002, described for the first time that zebrafish is able to regenerate up to ~20% of its heart ventricle after amputation, thus showing the most robust cardiac regenerative response in a vertebrate [188]. The injury leads to a blood clot formation that is subsequently replaced by fibrin and collagen to preserve the ventricular wall and seal the wound. From 7–9 days post-injury (dpi) to the next following weeks, this fibrotic tissue is replaced by new cardiomyocytes (CM). After 60 dpi the size and shape of the ventricle, as well as the heart beating contractile capability, gets back to normal [189]. The use of genetic fate-mapping approaches allowed establishing that the source of the new muscle cells is from pre-existing muscle cells, stimulated by injury to divide [190,191] but, it is not completely clear what molecular signals are involved in this process. Few insights came from a study of Sande-Melon et al. that identified a subset of sox10-positive cardiomyocytes within the uninjured heart with a high capacity to contribute to the new myocardium [192]. Ablation of these cardiomyocytes confirmed that they play an essential role during the heart regeneration.

The induction of CM proliferation is triggered and controlled by various cells and factors. The first responders to heart injury are inflammatory cells like neutrophils, macrophages, and T-cells [193,194,195]. The cryoinjury procedure revealed that early macrophage invasion, rapid appearance of angiogenic sprouts into the wound, and transient fibrosis are required for robust cardiomyocyte proliferation [182,196,197,198,199].

Several works assessed that the epicardium is involved in multiple aspects of cardiac repair after injury with the ability to regulate heart regeneration through secretion of soluble growth factors. Indeed, when activated, cells from the epicardium are able to proliferate and migrate to the injury area where they can secrete extracellular matrix components and molecules able to regulate cell proliferation and heart regeneration [189,200,201,202]. Furthermore, genetic ablation of the epicardium and its derivatives, disrupts CM proliferation and muscle regeneration, but the process renews following epicardium recovery [202].

Information available on the signaling pathways underlying cardiac regeneration in zebrafish is not very extensive but some of the identified factors, e.g., the Hippo pathway and its downstream effectors, the transcriptional co-activators Yes-associated protein (YAP) and transcriptional co-activator with PDZ-binding motif (TAZ), seems to be important in enhancing cardiac regeneration. Hereafter we summarize the main signaling pathways know and their specific functional involvement in cardiac regeneration.

The FGF family is fundamental in regeneration as they initiate a downstream signaling cascade through Ras/MAPK, Akt, and Stat signaling. Impaired heart regeneration was observed in blocked FGF-signaling transgenic fish [203]. Additionally, FGFs stimulate neovascularization and epicardial cell activation during the zebrafish heart regeneration [203,204]. However, the exact role of FGF ligands directly on zebrafish cardiomyocyte proliferation remains to be determined.

The Nrg1 is an extracellular ligand that also activates Ras/MAPK signaling. It is secreted from perivascular and epicardial cells at 7 dpi and has fundamental roles in regulating cardiomyocyte proliferation in both zebrafish and mice. Overexpression of Nrg1 increased cardiomyocytes proliferation after injury and inhibition of Nrg1 receptor caused reduction in cardiomyocyte proliferation after injury [179].

Growth factors are important modulators of zebrafish heart regeneration. The insulin growth factor (IGF) binds to receptors Igf1r and activates downstream signaling pathways that contribute to cell growth, differentiation, and anti-apoptotic pathways. Studies have shown that Igf signaling is a critical stimulator of cardiomyocyte proliferation [205]. Another growth factor, the platelet-derived growth factor (PDGF) is also important in cardiac regeneration as it activates downstream pathways involved in wound healing and proliferation. Studies have shown that PDGF ligands and receptors play an important role in heart regeneration and they are required in coronary vasculature formation during heart regeneration [206]. Apparently, their direct and main function in vivo is to support angiogenesis of the regenerating heart.

Transforming growth Factor-β (TGF-β) family ligands, such as TGF-β, BMPs, and activins seem to be key regulators in cardiomyocyte proliferation and scar formation. These factors operate through two different classes of receptors to phosphorylate distinct Smad transcription factors, which then complex with each other, and additional co-factors that regulate gene expression. Blocking TGF-β receptor activin Receptor-like Kinase 5/4 (Alk5/4) resulted with a significant decrease in pSmad3 and BrdU+ cardiomyocytes near the infarct suggesting that TGF-β is required for cardiomyocyte proliferation [207,208]. Further genetic loss-of-function mutations in activin A (*inhbaa*) showed a significant decrease in cardiomyocyte proliferation after cryoinjury [209].

BMP plays also an important role as chemical inhibition or overexpression of BMP-inhibitor *noggin3* delayed muscle repair, limited CM de-differentiation and cell cycle entry, while Induced global overexpression of *bmp2b* decreased the wound size [210].

Notch signaling pathway is also involved in heart development and cardiomyocyte maturation and proliferation. In zebrafish, following amputation of ventricular apex, Notch receptor expression becomes activated specifically in the endocardium and epicardium. Using a dominant-negative approach, Long Zhao et al. show the exquisite sensitivity of regenerative cardiomyocyte proliferation to perturbations in Notch signaling. They discovered that suppression of Notch signaling profoundly impairs cardiac regeneration and induces scar formation at the amputation site. Unexpectedly, hyperactivation of Notch signaling also suppressed cardiomyocyte proliferation and heart regeneration [211].

Additional regeneration effectors, such as miRNAs, are suggested being able to affect cardiomyocytes proliferation by inhibiting or activating the cell cycle [212]. Epigenetic regulation through chromatin remodeling or histone modification has also been shown to be involved in zebrafish heart regeneration [213] and seem to be important for cardiomyocyte regeneration.

### 11.3. Zebrafish as a Model of Human Regeneration

Zebrafish is a useful model for studying molecular mechanisms of regeneration [179] due to the availability of genetic data, fully sequenced genome, readiness for genetic manipulations and biosensor and reporter zebrafish lines [214,215]. In addition, the muscle structure and muscle-related gene expression are highly conserved between human and zebrafish and over 70% of human genes have a true ortholog in the zebrafish genome [216]. From a methodological viewpoint, many study on zebrafish regeneration employ larval stages whose transparent body allows easy tracking of structural changes during development and regeneration of the skeletal and heart muscle tissue.

Injured human heart does not regenerate and results in irreversible loss of myocardial cells. The damaged myocardium is replaced by fibrotic scar tissue that undermines pump function and leads to congestive heart failure and arrhythmia. Although several works suggest cardiomyocytes proliferation ability in the human heart [217], this process is not significant in response to injury [218] thus not providing a complete restoration of its functions. The zebrafish is probably one of the most important vertebrate model for studying heart developmental and regenerative properties relevant also to mammalian heart for its impressive regeneration ability following different forms of injury [187] that it is not based on stem cells or transdifferentiation of other cells but on the proliferation of preexisting cardiomyocytes [190,191]. Hence, studying the zebrafish model could expand the knowledge on cardiac regenerative processes and may contribute to the identification of specific molecules able to regulate the proliferation of these cells. This may provide insights for the design of future therapies for cardiac repair after myocardial infarction (MI) and other cardiac injuries in humans.

Concerning skeletal muscles, zebrafish and human share a high similarity at cellular, molecular, histological, and ultrastructural levels. In addition to the genetic tools for the expression of pathological phenotypes such as muscular dystrophy [219], both adult and larval zebrafish muscle have been shown to be a valuable models to study regeneration events by exploiting the possibility of performing selective injury of muscles while imaging morphogenetic processes using for example fluorescent reporter lines [178,220,221]. Notably, these studies also shed light on possible factors limiting mammalians regeneration abilities. Indeed, it has been shown that in order to allow a proper regeneration, and differently from mammals, zebrafish heart and skeletal muscles maintain the ability to activate specific gene regulatory networks (GNRs) in response to injury and perform epigenetic modifications necessary to trigger regeneration. In addition, a fundamental role is also played by the immune system whose harnessing has been shown to promote cardiac regeneration (for a review see [178]).

## 12. Mammals: Cell Therapy for Skeletal Muscle Regeneration

Mammalian skeletal muscle possesses a certain potential to regenerate, but this process can be compromised in several pathological conditions (e.g., neuromuscular disease, cancer-associated cachexia, or age-dependent sarcopenia) and following trauma the extended loss of muscle fibers cannot be fully recovered. These events lead to a weak regeneration and formation of fibrotic scar tissue, and result in loss of functional muscle mass. Consequently, the ability to perform intense muscular efforts and even easy, daily-life tasks may be impaired [222,223,224].

In the last decades, the scientific community devoted increasing efforts to develop therapies for the regeneration of damaged tissues in humans. This section aims at providing an overview of the cellular strategies that have been developed for improving skeletal muscle regeneration in mammals, particularly humans. As for the methods employed to study skeletal muscle regeneration, readers can refer to more comprehensive reviews [7,223,225,226,227].

Regenerative medicine aims at promoting the formation of new functional tissue by delivering precursor cells or bio-engineered tissue patches into the injured area. Indeed, therapeutic cells are isolated from the donor subject, expanded in culture (if needed), integrated into an acellular synthetic scaffold (in case of bio-engineered tissue patches), and transplanted into the recipient tissue. Although this general procedure looks simple, the choice of the specific therapeutic strategy is very complex due to the large number of different cell sources and implantation technologies. As for the cell source, the immunological compatibility between donor and recipient should be taken into account. Therefore, in the clinical setting, autologous transplantation is often preferred over the heterologous or xenologous one [223].

Focusing on skeletal muscle regeneration, the following types of cells have been employed: satellite cells, muscle-derived stem cells, myoblasts, mesoangioblasts, hTERT/Bmi-1- or hTERT/CD4-immortalized muscle precursor cells, pericytes, CD133+ cells, hematopoietic stem cells, mesenchymal stem cells, myoendothelial cells, side-population interstitial cells, myogenic precursor cells, dental pulp pluripotent-like stem cells, and eventually induced pluripotent stem cells (iPSCs) [228,229]. In mammalian animal models, these cells demonstrated a certain ability to proliferate both in vitro and in vivo, and to generate functional, integrated skeletal muscle tissue [228,230]. However, the ideal source of myogenic cells is still debated, due to a number of limitations such as the availability of bioptic biological material, the tumorigenic risk of immortalized cells, the capacity of cells to proliferate and graft into the host tissue, etc.

The possibility to artificially reprogram fully differentiated mammalian cells into iPSCs provides a virtually unlimited source of pluripotent stem cells for almost every individual. Nowadays, iPSCs represent a very useful tool for developing patient-specific regenerative therapies, thanks to the effective and reliable protocols for the expansion and differentiation of these cells both in vitro and in vivo [231,232,233]. Moreover, the opportunity to generate myoblasts, as well as different types of cells from iPSCs, such as neurons, endothelial cells, pericytes, and to edit their genome through CRISPR/CAS9 and TALEN technologies, further enhances their possible use for therapeutic applications [234,235]. IPSCs are stem-like cells generated by the reprogramming of fully differentiated somatic cells. Reprogramming strategies involve either the delivery of genetic material encoding reprogramming factors (e.g., Oct4, Sox2, Nanog, c-Myc, Klf4, and Lin28) or the administration of specific miRNAs or cocktail of proteins and small molecules into the somatic cells. In the latter case, reprogramming is triggered by direct activation of the endogenous stem-cell factors. The integration of exogenous DNA into the host genome relies on retroviruses, lentiviruses, and piggy Bac transposons; the non-integrating DNA and RNA procedures use adenoviruses, Sendai virus, plasmids, episomal vectors, and mRNA. Once generated, iPSCs can proliferate indefinitely in culture and differentiate into any kind of adult somatic cell by administering the appropriate growth or differentiation factors. In particular, the induction of myogenesis can be achieved either by expressing exogenous muscle-specific transcription factors, such as MYOD, PAX7, and PAX3, into the cells, or by activating endogenous pro-myogenic differentiation pathways (e.g., Wnt and BMPs signaling) by supplying, in the culture media, specific molecules, such as GSK3b inhibitors, bFGF, FGF-2, epidermal growth factor (EGF), DAPT, forskolin, BMP inhibitors, hepatocyte growth factor (HGF), and IGF-1 [236].

From a clinical perspective, the use of genetic manipulation, although more effective, is not safe, due to the risk of genetic recombination. Methods based on the supplementation in the culture media of chemical compounds prompting cell reprogramming and differentiation toward a skeletal muscle phenotype, are preferred. It should also be considered that in vitro, the differentiation process has not a 100% efficiency. Therefore, besides myogenic cells, other cell types, such as neural cells and fibroblasts, originate within the culture. In addition, myogenic cell at different stages of differentiation coexist in the entire cell population and a cell sorting strategy (by means of a fluorescence-activated cell sorter) is required to obtain a pure pool of myogenic precursor cells [237,238,239]. The selected population of progenitor cells can be transplanted into the host via different methods, such as intramuscular injection, systemic cell delivery, and microsurgical implants. Each of these methods has pros and cons and a unique optimal strategy has not been defined yet. Indeed, systemic delivery results in a highly variable success rate, intramuscular injection requires multiple local treatments, and implants involve more invasive procedures.

The efficacy of these regenerative therapies strictly depends on the capacity of the transplanted cells to engraft the injured site, survive, proliferate, differentiate, and integrate within the native skeletal muscles. To promote the success of these therapies, specific strategies have been developed: artificial co-administration of small molecules and growth factors such as TGF-b and myostatin inhibitors, IGF-I, fibrin, keratin, collagen; tissue engineering and bioprinting for the generation of synthetic scaffolds embedding myogenic cells [228]. The composition and the three-dimensional architecture of the synthetic scaffold provide structural and functional support to the cells and promote the formation of new, functional skeletal muscle tissue, as well as the establishment of a pool of self-renewing stem cells [240,241,242,243,244].

Although regenerative medicine applied to muscle disorders has greatly advanced, a standard for cell-based therapeutic interventions is still lacking. At the moment, we are far away from effective treatments promoting skeletal muscle regeneration in mammals. However, cell therapies, benefiting from the great potential of iPSCs, genome editing, tissue engineering, and cell biology methods, hold great promise for successful skeletal muscle regeneration.

## 13. Conclusions and Future Perspectives

Model organisms have always been playing a fundamental role to uncover general biological mechanisms common also to humans and historically they have a key role in translational medicine. In the last decades, deeper mechanistic studies of animal diversity have been made possible by the availability of a broader and affordable toolbox of technical resources such as genomics, transcriptomic, connectomics, and many other molecular biology techniques [245]. One emblematic example is the introduction of CRISPR/Cas9 genome editing, which made functional approaches possible in a wider range of so-called non-canonical model organisms. These techniques are allowing scientists to address a broader spectrum of biological problems exploiting the diversity of animal biology [246]. For instance, “simple” model organisms like Planarians and Echinoderms greatly benefited from these advancements and are nowadays considered *bona fide* models in translational research for the possibility of performing cell tracking and expression profiling of their tissue.

Nonetheless, the number and variety of animal species currently used in biomedical research are still rather limited. The reason is certainly not the suitability of particular species to a specific scientific question, but rather to the laboratory amenability (due for example to the flexibility and cost of breeding, the animal availability, the length of life cycles, the optical transparency, the possibility to perform genetic manipulations, etc.) and the familiarity of a critical mass of researchers with established animal models. The result is a scenario where only a few species retain a legitimate biomedical interest, while many others are left aside.

Nowadays, only few animal models (mainly rodents, chicken, and to some extent zebrafish) have been suitably exploited in muscle regeneration studies and various injury protocols (e.g., surgery, chemically induced muscle damage, genetic ablation, denervation-devascularization, intensive exercise, etc.) have been employed [178,225,226,247,248,249]. Except for zebrafish, none of the animal species presented in this review have been systematically tested for the efficacy and utility of the diverse injury models; indeed, the main methods employed to stimulate regeneration are based on physically- (by surgery or irradiation) and chemically-induced muscle damage, and genetic ablation.

It is important to point out that the application of a specific injury protocol in different animal species, given their diversity in body morphology, physiology and regeneration mechanisms, may not lead to directly comparable results. Thus, a comparative experimental approach taking into account animal diversity is fundamental to explain the variation in regenerative capacity through phylogeny, ontogeny, and even aging [250]. This may provide hallmarks of molecular homology between regenerative events in the metazoans [251] as well as an explanation of the loss or restriction of regeneration abilities occurring in some animals like *D. melanogaster* and *C. elegans*. Notably, even with their limited regeneration abilities, these animals may supply important insights into the negative regulation of this supposedly advantageous attribute.

In this review, we wished to provide the researchers interested in muscle regeneration with a lookout of muscle diversity across animals, and a prospect on their regenerating potentials (see Table 1). This work was not meant to provide ‘guidelines’ into animal selection when addressing specific regeneration issue but to offer ‘insights’ on open questions and new standpoints. We thus gave an overview of how cell precursors and regeneration strategies are adopted to partially or completely restore muscular components in various clades or even in different species within a single clade.

We showed that the presence and nature of cell precursors giving rise to new muscles have been addressed in most of the clades and seem to be rather heterogeneous although a proper cell fate mapping has in many cases not yet been disclosed. In Tunicates, the sister-group of vertebrates, we saw the involvement of putative pluri- or multipotent stem cells during regenerative processes, and the partial co-option of embryonic myogenesis (the latter highly conserved in vertebrates) during muscle regeneration.

We saw that cell migration and tissue re-organization are crucial for regeneration and, given their rather ‘simplicity’, animals like Porifera, Cnidarians, and Planarians may represent valuable model to investigate these aspects. Moreover, we saw that animals use different regeneration strategies, e.g., epimorphosis and morphallaxis, that are equally successful and that may also coexist within the same organism, as it happens in amphioxus, to meet tissue specific regeneration requirements. From a translational perspective, these examples reinforce the idea that regenerative medicine should not seek out a ‘regeneration blueprint’ but rather a set of ‘context-dependent’ regeneration strategies.

We highlighted how the gene regulation aspect has been studied to various extents in many clades and is particularly well assessed for Echinoderms. On the other hand, the range of epigenetic controls has been investigated only in few species, such as zebrafish. Indeed, epigenetic studies are still at their dawn in non-vertebrates.

Comparing the architectures of a “regeneration permissive” vs. a “non-permissive” gene regulatory network (GRN) between closely related species, or finding signatures of epigenetic control of regeneration can fuel the expanding field of synthetic biology or allow for an ample testing of new classes of drug, targeting molecular and cellular mechanisms conserved in human but more functionally testable in other animal models. In this sense, regenerative therapies might greatly benefit from these comparative studies.

We also showed that, besides genetics and epigenetics, another key factor for a successful regeneration in metazoans is the ‘environmental qualification’, i.e., the extracellular matrix (ECM) structure, remodeling, composition, collagen content, cytoarchitecture, and secreted factors influencing the heterogeneous population of cells present within the regenerating environment [2,223]. Environmental qualification is currently considered a fundamental aspect of cell engraftment during transplantation and the lack of knowledge on this topic represent one of the current bottlenecks in regenerative medicine. Indeed, to improve regeneration of muscle tissues, transplantation of cells can be done through scaffolds ideally mimicking native tissues. Scaffolds can be made by natural polymers, synthetic polymers, or even decellularized ECM that can be filled after implantation by stem cells to restore muscle morphology. They are used to provide chemical and physical cues to transplanted cells and to create a microenvironment niche that favor survival of the resident cells and engraftment of the transplanted cells [223]. These study are at the forefront of tissue engineering and aim at providing a structural and biochemical framework for regeneration. In this regard, animals such as octopus and zebrafish can be useful models to study the cytological and histological architecture of the regenerating environment thus providing information on how to model and enrich the regenerating niche [109,189,251].

From a more clinical perspective, the information provided by studying and comparing different animal models, without forgetting their phylogenetic framework, can help to address the problem of lack of regeneration in human tissues and might eventually be used to overcome the limits of muscle regenerative therapy.

## Figures and Tables

**Table 1:** Overview of muscle cell types and regenerative potentials of the animals treated in this review. * Muscle type discussed in this review in the context of regeneration, ** Induced pluripotent stem cells, can be derived from various species, *** Mostly repair.

Animal Species	Muscle Type *	REG	Cell Precursor	Known Signaling Pathways and Molecular Players
IPSC **	Any	n/a	n/a	MyoD, Pax7/3, Wnt and BMPs
**Vertebrates**	Striated; Cardiac	yes	Satellite stem cell; Pre-existing muscle cells	Notch, BMP, TGF-β, IGF, FGF family, Ngr1; Pax3/7, Met
**Tunicates**	Striated, smooth-like, cardiac	yes	Stem cells-like precursors (?)	Notch, Nk4, Tbx1/10, MRF
**Cephalocordates**	Striated, mononucleated	yes	De-differentiation, Multipotent cells	Pax3/7, Wnt/β-catenin and BMP
**Echinoderms**	Smooth-like, striated, mononucleated	yes	De-differentiation	BMP/TGFB, HOX, Ependymin, etc.
**Arthropods**	Striated	no ***	Adult muscle precursor (AMP)	Notch-Delta, Transcription factor Zhf1
**Nematodes**	Striated, mononucleated	no	---	---
**Mollusks**	Striated, mononucleated	yes	Sarcoblasts (?)	AChE, Growth factors (EGF, FGFs and VEGF)??
**Platyhelmintes**	Combine features of both vertebrate skeletal and smooth muscle cells	yes	Neoblasts: Adult pluripotent stem cells	Many known signaling pathways such as PCGs, Wnt/β-catenin, FGF family, insulin/IGF-1, Pax3/7, TGF-β, Hox genes, etc. (see text for references)
**Cnidarians**	Epitheliomuscular, smooth	yes	Hydrozoan: i-cells in Hydractinia, epithelial stem cells in Hydra Anthozoan: yet to be determined	The myogenic gene repertoire is present in cnidarians, but no experimental evidence relate them to the myogenic trajectory
**Porifera**	Myocytes	yes	Adult stem cells (ASC) Totypotent, pluripotent cells	ADPRC (ADP-ribosyl cyclase), TGF-β
